# Production of multimodal signals to assert social dominance in white-lipped peccary (*Tayassu pecari*)

**DOI:** 10.1371/journal.pone.0280728

**Published:** 2023-02-24

**Authors:** Raimundo N. Alencar, Sérgio L. G. Nogueira-Filho, Selene S. C. Nogueira

**Affiliations:** Laboratório de Etologia Aplicada, Universidade Estadual Santa Cruz, Ilhéus, Bahia, Brazil; McGill University, CANADA

## Abstract

In this study we aimed to examine whether the ‘redundancy’ (a backup function to ensure the signal transmission) or ‘multiple messages’ (sensory communication system in combination) hypothesis would explain the function of multimodal communication of white-lipped peccaries (*Tayassu pecari*–WLPs). We also aimed to assess the individual factors (the social rank and sex of the sender) influencing the production of, and responses to unimodal and multimodal signals. We determined the social rank of 21 WLPs living in two captive groups and quantified the production of unimodal and multimodal signals when displaying threatening and submissive behaviors. WLPs most often produce multimodal signals independent of a previous unimodal signal failure, which suggests that they were adding more information, such as the sender’s size, rather than merely increasing efficacy by engaging a different receiver’s sensory channel. There was no effect of the sender’s sex in the production of, and responses to, multimodal signals. However, the higher the sender’s social rank, the greater the production of multimodal signals when WLPs were displaying threatening behaviors; whereas the lower the sender’s social rank, the greater the production of multimodal signals when displaying submission behaviors. Multimodal signals elicited more non-aggressive responses than did the unimodal signals when displaying a threat. Moreover, the higher the sender’s social rank, the greater the occurrence of non-aggressive responses to multimodal signals when displaying a threat; whereas the opposite occurred when displaying submission. Our findings support the ‘multiple messages’ hypothesis to explain the function of multimodal signaling during agonistic interactions in WLPs. Additionally, both the production of, and responses to, multimodal signals are related to the sender’s social rank. These results allow us to suggest that the production of multimodal signals may have a key role in mitigating conflict and thus promoting group cohesion among white-lipped peccaries.

## Introduction

Animal communication occurs through the use of signals or a combination of them, coming from distinct sensory channels [1–3]. When sending a signal that engages only one sensory channel of the receiver (visual, auditory or tactile), the communication is named unimodal. However, when the sender emits signals that engage the receiver’s sensory communication system in combination, the communication is named multimodal [2,4]. Each sensory channel can also have a backup function and so ensure the signal transmission–the ‘redundant signals’ hypothesis [4–8]. Alternatively, by providing different messages through different sensory signals, the use of multiple signals can allow the transfer of independent information, such as the size of the sender, leading to an increase in the information content that is sent to the receiver–the ‘multiple messages’ or ‘complementarity’ hypothesis [2,9–14]. For additional hypotheses that have been proposed regarding multimodal communication functions, see Fröhlich and van Schaik [11],

Group-living individuals may show a wide variety of signals in their communication repertoire [15,16]. In relatively stable societies composed of many members, the potential for interactions to occur increases, whether these interactions are cooperative or aggressive. The ‘social complexity hypothesis for communicative complexity’ states that the greater the social diversity of these groups, the greater their signaling system [15,17–19]. This is because individuals need to send a greater variety of signals to make the recognition of message less misunderstood, such as when a chimpanzee (*Pan troglodytes*) indicates to other group members its grooming needs by over-scratching [20]. Another possibility is to use a multimodal signal [1,2] as happens with *Gymnorhina tibicen*, a social bird that can signal the presence of a predator to conspecifics simultaneously using alarm calls and pointing its beak towards the threat [21]. During conflicts, the message must be quickly understood; thus the individuals can use threatening displays, especially those that emphasize the body size and fighting ability of the sender, to intimidate the receiver [12,22]. Therefore, the engagement by the sender of more than one sensory communication channel of the receiver seems to play an important role in the secure interpretation of the information (e.g. Sumatran orangutan, *Pongo abeli* and Bornean orangutan, *P*. *pygmaeus* [23]; bonobo, *Pan paniscus* [13], and chimpanzee, *P*. *troglodytes* [24,25]), as well as in partner selection [3], social group dynamics [16], and adaptation of individuals to environments with higher or lower risk (e.g. lowland paca, *Cuniculus paca* [18]). However, from these studies, only a few have analyzed the relationship between multimodal communication usage and the assertion of the status of the sender [24–27]. Therefore, to clarify the communicative and social function of multiple signal emission, it is necessary to systematically study the use of multimodal communication for signaling the dominance hierarchy position, hereafter referred to as social rank.

The competition for limited resources can generate conflict among group members, making it necessary to use mechanisms that prevent the intensification of aggressive behavior, such as dominance hierarchy [28–30]. Dominance hierarchy is a common mechanism in group-living species [31–35], in which dominant individuals have priority access to limited resources to minimize conflicts within the group caused by competition and thus avoid fights that cause considerable waste of time and energy, as well as a risky of injury and group dispersion [36,37]. In general, there is a consensus that dominance is associated with individual physical attributes, such as horn size (e.g. reindeer, *Rangifer tarandus* [38] and woodland caribou, *R*. *tarandus caribou* [39], prior experience (winner-loser effects, e.g. *Sus scrofa [32]* or is associated with the individual testosterone titers (e.g. mouse *Mus musculus* [40]); for a full review see Tibbets et al. [41]. However, little is known about behavioral factors related to the maintenance of dominance hierarchies [42], such as the possibility of using multimodal communication to signal the sender’s social rank. This issue can be thoroughly investigated in white-lipped peccaries (*Tayassu pecari*) [43].

White-lipped peccaries (WLPs) are distributed from Mexico to Northern Argentina [44], where they primarily inhabit humid tropical forest, predominantly consuming palm fruits [45–48]. The species, which shows no sexual dimorphism [44], lives in mixed-sex herds that are relatively stable and often composed of hundreds of individuals in the Amazon region [48,49]. However, due to seasonal food shortages, groups may split into smaller subunits, ranging from 27 to 70 individuals [48,50]. Moreover, groups of as few as five to 16 individuals have also been observed in overhunted areas [48,51,52]. In the wild, the group-living style is an important anti-predator strategy for WLPs, which collectively can counterattack predators such as jaguar (*Panthera onca*) and puma (*Felis concolor*) [44]. Thus, it seems that the social behavior of the WLPs has evolved to minimize conflicts within the group and promote its cohesion [53]. One of these social strategies is the linear dominance hierarchy reported for captive WLPs, which is unique and encompasses both males and females [53–56]. Additionally, during disputes, WLPs show body postures that indicate threat and submission [54–56], together with the grunt call, which is modulated by the sender to reinforce its subordination to a dominant animal [43]. Moreover, the species is characterized by a dorsal gland, present in both males and females and that plays a role in olfactory communication [44]. Furthermore, WLPs live in groups with comparatively greater social complexity, similar to that observed in cognitively sophisticated non-human primates, which can be exemplified by the occurrences of third-party conflict interventions [53] and social play interactions involving not only young ones but also adults [56].

Therefore, we aimed in the current study to examine whether the ‘redundancy’ or ‘multiple messages’ hypothesis would explain the function of multimodal signaling by WLPs during agonistic interactions. Based on the ‘redundant signals’ hypothesis [4,5,8], following Hobaiter et al. [24] we expected that WLPs would produce a multimodal signal more frequently after a failure of a unimodal signal. Alternatively, based on the ‘multiple messages’ hypothesis [9,10,13,14], if by emitting multiple signals the WLPs are adding more information (e.g. the size/body mass and/or age of the sender and its intention) rather than merely increasing efficacy, as verified among wild chimpanzees [24] and captive bonobos [57], we expected that WLPs would produce more frequently a multimodal signal independent of the failure of an initial unimodal signal.

We also aimed to assess the individual factors influencing the production of, and responses to, unimodal and multimodal signals. Due to the use of body postures in combination with modulation of grunt call to reinforce its subordination to a dominant individual [43], we expected that WLPs with more submissive social status would produce more multimodal signals during disputes for limited resources, such as food. Additionally, because there is a lack of difference in aggressiveness between males and females, and threatening and submissive behavioral patterns also do not differ between the sexes [54,55], we predicted no difference in the production of multimodal signals between males and females. Furthermore, due to the considerable costs, in terms of time and energy expenditure [4,5,12], we expected that the response to multimodal signaling of either threatening or submissive behaviors would be more non-aggressive interactions in comparison with the response to unimodal signaling, regardless of the sender’s social rank or sex.

## Methods

### Ethics statement

This work followed the Brazilian laws and was approved by the Animal Use Ethics Committee (CEUA) of the Universidade Estadual de Santa Cruz-UESC (protocol # 01/2019). Additionally, the scientific wild animals breeding center of UESC, used in this study, was registered at the Brazilian Environmental Agency (IBAMA #1/29/2001/00022-7).

### Study site and animals

We observed 21 adult white-lipped peccaries (WLPs): 12 females and nine males, living in two groups maintained in two out-door facilities of the Laboratório de Etologia Aplicada (Labet), located at Universidade Estadual de Santa Cruz, Ilhéus, Bahia, Brazil (14°47’39.8” S, 39°10’27.7” W). Group 1 (G1) was composed of 13 WLPs (eight females and five males), housed in a 451 m^2^ paddock. Group 2 (G2) was composed of eight WLPs (four females and four males), housed in a 400 m^2^ paddock. The two paddocks were not contiguous, with 20m of distance between them. Both paddocks had a dirt floor, bushes and several trees that provided natural shade. All WLPs were born and raised in captivity, were aged between 3 and 10 years old, and were identified with plastic ear tags (6.0 cm x 4.5 cm) of different shapes. Thirty days before the beginning of the behavioral observations, we weighed the animals and started a habituation process to minimize observer effects on WLPs’ behavior.

During the study, food was provided twice a day at 8:00 am and 4:00 pm, totaling 700 g in dry matter basis per animal and day. The diet was composed of a mixture of corn grain and soybean meal mixed with mineral salt, furnishing 120 g/kg of gross protein and 14.5 MJ/kg of digestible energy in dry matter, which meets the nutritional needs for WLP adults [58]. Seasonal fruits and Napier grass (*Pennisetum purpureum)* were occasionally provided. The caretaker offered the food in feeders distributed in the proportion of one feeder for every three animals, to reduce the occurrence of food disputes (SLGNF, unpublished work), while water was available *ad libitum* from a concrete drinking fountain.

### Data collection

For data collection, an observer was positioned approximately 5 m from the fence, and this person video-recorded (JVC camcorder, GZ-HD500, Tokyo—Japan) the animals for subsequent analysis. The animals were observed for 60 non-consecutive days (January to March, 2019) between 8:00 am and 9:00 am and between 4:00 pm and 5:00 pm, which corresponds to feeding periods. We chose this observation period because the highest numbers of disputes usually occur during feeding, and thus we could observe disruptions in social stability more often [54]. Using the continuous focal animal sampling method [59], we coded the threatening and submissive behaviors (details below) to determine the individuals’ social rank in the dominance hierarchy of each group. Each focal observation lasted 10 min/animal, totalizing 38.5 hours of data collected. We randomized the observation of groups G1 and G2, and attained 110 minutes of data collection per individual with the same focal number in the morning and in the afternoon. We finished the observations when at least three dyadic agonistic interactions with a clear outcome, i.e. winners and losers clearly recognized, between all possible dyads of each group, had been recorded.

We also coded the production of unimodal and multimodal signals during agonistic interactions when WLPs were displaying threatening and submissive behaviors. The observers identified the sender and the receiver of the communication acts, the behavior displayed (threatening or submissive), the engagement of the receiver’s sensory communication channels (visual, sonorous or tactile), either alone (unimodal) or in combination of two or more than two receiver channels (multimodal) as well as its temporal occurrence either as after a unimodal signal failure or without a previous failure. Furthermore, we coded the receivers’ responses to unimodal and multimodal signals into a dichotomous “non-aggressive/engagement” category as well. The receiver could have either emitted a soft, non-aggressive interaction (non-aggressive response), like standing or even eating next to the sender; or it may have issued a dominance-submissive behavior response (engagement response), such as pushing or reclining (Table 2). We excluded from the analyses all interactions that could not determine with certainty which sensory channels of the receiver (visual—V, auditory—S, and tactile—T) were engaged.

Only R.N.A.J. collected the data, but the coding of all visible agonistic encounters between group members in the 38.5 h of video-recorded images was equally split between S.L.G.N.F. and S.S.C.N. Following Leonardo et al. [53], for each dyadic agonistic interaction the observers recorded: the type of conflict (decided–with a clear outcome–or inconclusive outcome); winner and loser identities of decided agonistic interactions. We employed the Kendall’s concordance coefficient (*W*) for 10% of the video-recorded images to assess the inter-observer reliability of coding, which were relatively high, for the following variables: winner and loser identities of decided agonistic interactions (*W* = 0.92), signal type (unimodal or multimodal) (*W* = 0.95), behavior displayed (threatening or submissive) (*W* = 0.96), and receiver response (non-aggressive response or engagement) (*W* = 0.95).

### Data and statistical analyses

To analyze the social structure, we selected only agonistic interactions in which the winner and loser were clearly defined, after disregarding interactions (N = 23) in which the outcome of the dispute was not clear. We analyzed these data using the software package SocProg 2.9 [58] to determine the Landau corrected linearity index (*h’*) [60]. This index ranges from zero (non-linear hierarchy) to 1.0 (complete linear hierarchy), and the statistical significance of *h*’ is provided by a re-sampling procedure using 1000 randomizations. The same software released the directional consistency index (DCI), which measures the direction of dominance within the hierarchy and also ranges from zero (equal exchange of dominance acts) to 1.0 (complete uni-directionality) [61]. Thereafter, we calculated modified David’s scores (MDS) [62], to determine the individuals’ social rank in the dominance hierarchy of each group using DomiCalc v. 1.0 software [63]. Thereafter, we compared the body mass, age, and MDS between females and females using the Students *t*-Test (*t*-Test). Following, we tested the possible associations between the individuals’ body mass, age and their social rank using the Pearson correlation test in each group. We performed these analyses after checking the normality of these variables using the Anderson-Darling test.

To test whether the ‘redundancy’ or ‘multiple messages’ hypothesis explains the function of multimodal signaling during agonistic interactions, we compared the production of multimodal signals according to the temporal occurrence through generalized linear mixed models (GLMMs). We constructed one model per behavior displayed (threatening and submissive) and each model included the temporal occurrence (after a unimodal signal failure and without a previous failure) and the sex of the sender as fixed factors, the senders’ social rank (MDS) as co-variable, and all their possible interactions, followed by *post hoc* Tukey tests and linear regression analyses when appropriate. The identity of WLPs nested within their group was included as a random factor in both models. This allowed us to control for repeated measurements and dependencies. We also graphically checked the residuals of every model for normal distribution and homoscedasticity, and all data were log-transformed to satisfy these assumptions.

We also constructed GLMMs–one model per signal type produced (unimodal or multimodal)–to assess the relationship between unimodal and multimodal signaling and the sender’s social rank and/or sex. The models included the behavior displayed (threatening and submissive) and the sex of the sender as fixed factors, the sender’s social rank (MDS) as co-variable, and all their possible interactions, followed by *post hoc* Tukey tests or linear regression analyses when appropriate. The identity of WLPs nested within their groups was included as a random factor in the models. As commented before, this allowed us to control for repeated measurements and dependencies. We graphically checked the residuals of every model for normal distribution and homoscedasticity, and all data were log-transformed to satisfy these assumptions.

Following Pollick and de Waal [57], we used Wilcoxon signed-ranks tests to evaluate whether multimodal signal production was more effective in eliciting a non-aggressive response compared to unimodal signaling during agonistic interactions. Thereafter, to test the prediction of lack of relationship between the senders’ sex and social rank and the responses to multimodal signaling we used GLMMs to compare the non-aggressive and engagement responses to multimodal signaling when WLPs were displaying threatening or submissive behaviors (one model per behavioral pattern), following Wilkie et al. [64]. The models included the signal type (unimodal or multimodal) and the sex of the sender as fixed factors, the MDS as co-variable, and all their possible interactions, followed by *post hoc* Tukey tests and linear regression analyses when appropriate. We also included the identity of WLPs nested within their group as a random factor in both models. As commented before, this allowed us to control for repeated measurements and dependencies. We used the software Minitab 19.1 (Minitab Inc., State College, PA) and set the significance level at α = 0.05 for all analyses.

## Results

### Social structure analysis

From the analysis of the clearly decided dyadic agonistic interactions in both groups (G1 = 225 and G2 = 184), we determined highly linear hierarchies (G1: *h’* = 0.82, *P* < 0.001; G2: *h’* = 0.96, *P* = 0.001). Additionally, the directional consistency indexes (DCI) were 1.0 in G1 and G2, meaning a complete uni-directionality of dominance within the hierarchy in both groups. Moreover, the individuals’ social rank (MDS) showed that in both groups one male was the top-ranking individual (highest MDS) followed by two (G1) or three females (G2), while the lowest social ranks were occupied by three males in the group G1 or by two males and one female in the group G2 (Table 1).

**Table 1:** Sex, age, body mass and social rank (modified dominance David’s scores–MDS) of the individuals of two captive white-lipped peccary groups (G1: N = 13; G2: N = 8).

G1					G2				
Code	Sex	Age (years)	Body mass (kg)	MDS	Code	Sex	Age (years)	Body mass (kg)	MDS
M1	Male	7.9	44.0	63.6	M1	Male	6.2	40.6	23.9
F2	Female	9.5	40.9	47.0	F2	Female	7.2	40	16.0
F3	Female	5.9	37.8	39.3	F3	Female	4.6	38.8	8.2
M4	Male	8.3	40.0	11.8	F4	Female	7.0	37.7	-2.2
F5	Female	7.4	35.8	13.4	M5	Male	4.6	39.8	-5.1
F6	Female	7.2	39.8	-3.7	M6	Male	6.4	35.9	-10.3
F7	Female	3.8	36.8	-10.9	F7	Female	4.6	35.8	-13.1
M8	Male	8.7	35.8	-9.6	M8	Male	7.6	35.5	-17.4
M9	Male	7.6	35.3	-19.5					
M10	Male	6.1	33.8	-21.2					
M11	Male	5.6	32.0	-24.6					
M12	Male	4.8	35.8	-41.8					
M13	Male	7.2	29.3	-43.2					

MDS did not differ between females and males in both groups (G1: female: mean = 17.3, standard error (SE) = 11.3, *N* = 5; male: mean = -10.7, SE = 12.3, *N* = 8, *t*-test = 1.68, *P* = 0.124; G2: female: mean = 2.25, SE = 6.3, *N* = 4; male: mean = -2.5, SE = 9.1, *N* = 4, *t*-test = 0.41, *P* = 0.701, Table 1). Body mass did not differ between females and males in both groups (G1: female: mean = 38.0 kg, standard error (SE) = 1.0, *N* = 5; male: mean = 35.9 kg, SE = 1.6, *N* = 8, *t*-test = 1.12, *P* = 0.290; G2: female: mean = 38.0 kg, SE = 0.9, *N* = 4; male: mean = 38.0 kg, SE = 1.3, *N* = 4, *t*-test = 0.01, *P* = 0.940, Table 1). Age also did not differ between females and males in both groups (G1: female: mean = 7.7 kg, SE = 0.6, *N* = 5; male: mean = 6.4 kg, SE = 0.6, *N* = 8, *t*-test = 1.60, *P* = 0.147; G2: female: mean = 5.8 kg, SE = 0.7, *N* = 4; male: mean = 6.2 kg, SE = 0.6, *N* = 4, *t*-test = -0.37, *P* = 0.728, Table 1). We recorded statistically significant correlations between individuals’ body mass and their MDS in both groups (G1: *r*_*Pearson*_ = 0.83, *N* = 13, *P* < 0.0001; G2: *r*_*Pearson*_ = 0.87, *N* = 8, *P* < 0.0001). However, there were no significant correlations between an individual’s age and its MDS in both groups (G1: *r*_*Pearson*_ = 0.46, *N* = 13, *P* = 0.113; G2: *r*_*Pearson*_ = 0.03, *N* = 8, *P* = 0.936). There were also no significant correlations between the individual’s age and its body mass in both groups (G1: *r*_*Pearson*_ = 0.37, *N* = 13, *P* = 0.218; G2: *r*_*Pearson*_ = -0.15, *N* = 8, *P* = 0.728).

## Redundancy *versus* multiple messages hypothesis

During the observations, we recorded 408 agonistic interactions in both groups, which involved the display of 208 threatening and 200 submissive acts in which it was possible to identify the production of signals that engage the receiver’s channels (visual, auditory, and tactile) either alone (unimodal) or in combination (multimodal) (Table 2). WLPs produced most of the unimodal signals (86.8%) using only the visual channel of the receiver. They produced most of the multimodal signals (85.6%) when displaying a threat; of which in almost half (45.3%) they used the combination of visual and auditory channels of the receiver; followed by the combination of visual, auditory, and tactile channels (28.4%) and visual and tactile channels (26.3%) (Table 2). However, for the totality (100%) of the multimodal signals produced when displaying submission, the WLPs used the combination of visual, auditory, and tactile channels of the receiver (Table 2).

**Table 2:** Number of times each signal type (unimodal or multimodal), engaging different sensory channels of the receiver (visual- V, auditory- A, and tactile—T), was produced by white-lipped peccaries when displaying threatening and submissive behaviors.

Number of times	Signal type (sensory channel)	Behavior (threatening or submissive)	Description*
131	Unimodal (V)	Face (threatening)	An animal, approached by or having approached another, with erect dorsal bristles, stood with all four legs squarely planted, and the head held level. The head was not lowered, and the ears may or may not have been held back.
20	Unimodal (V)	Lower head (submissive)	The individual approached by or having approached another, lowers its head with its ears turned back.
86	Multimodal (V and A)	Facing while vocalizing (threatening)	The individual raises its head to face another, raising dorsal bristles and vigorously exhaling air through the snout or emitting a snarl and/or a vocalization similar to intense snoring.
50	Multimodal (V and T)	Push (threatening)	An animal shoves another, most frequently with its shoulder, and more rarely with its head.
35	Multimodal (V and A)	Lower head while vocalizing (submissive)	An individual lowers its head with its ears turned back while emitting the grunt call.
54	Multimodal (V, A, and T)	Facing while vocalizing, and pushing the opponent (threatening)	An individual raises its head with erect dorsal bristles, pushes the opponent while emitting a snarl and/or a vocalization similar to intense snoring.
32	Multimodal (V, A, and T)	Reclining while vocalizing and touching the opponent (submissive)	An individual lowers its head or reclines, kneeling on its forelimbs emitting grunt calls and touching the opponent’s chin with its head or snout.

*Descriptions modified from [55,56,65].

**Fig 1:**
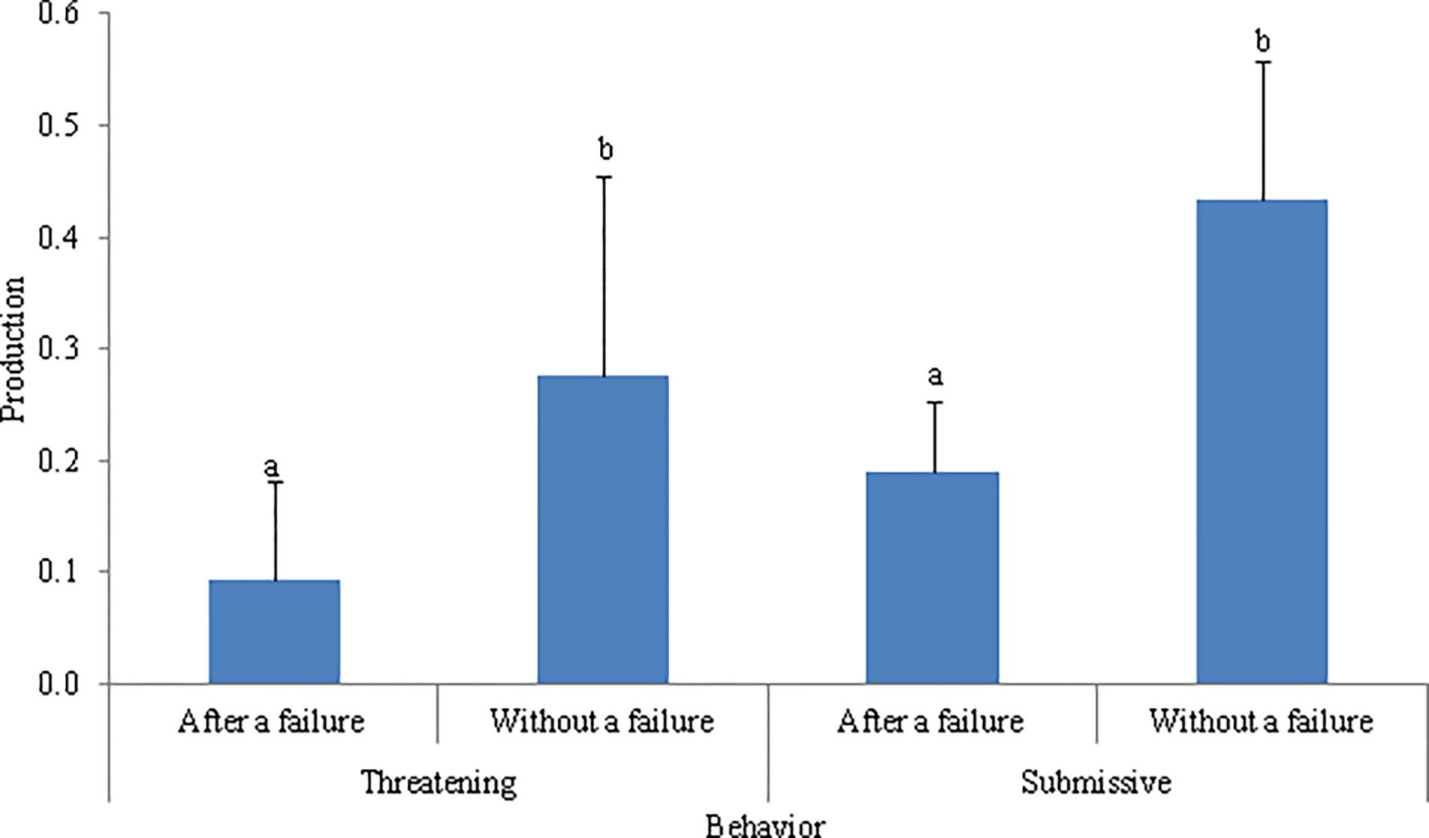
Mean (log-transformed) production of multimodal signals by white-lipped peccaries (N = 21). Animals may be displaying threatening or submissive behavior according to temporal occurrence. Production occurred either after a unimodal signal failure or without a previous unimodal signal failure. Error bars denote SE of means. Different letters above bars within the same behavior represent a significant difference (*P* < 0.05).

The temporal occurrence affected the production of multimodal signals when WLPs were displaying threatening behaviors (F_1, 17.0_ = 7.77, *P* = 0.013) and submissive behaviors (F_1, 17.0_ = 8.38, *P* = 0.010) (Table 3). The *post hoc* Tukey tests showed that WLPs more often produced multimodal signals without a previous unimodal signal failure than following a failure when displaying both threatening behaviors and submissive behaviors (Fig 1).

**Table 3:** Effect of the senders’ social rank (modified dominance David’s scores–MDS), sex, and temporal occurrence (after failure using unimodal signal or independent of a previous failure) in the production of unimodal and multimodal signals by white-lipped peccaries (N = 21).

Term	DF Num*	DF Den*	F Value	*P*
Threatening				
MDS	1.00	16.14	58.93	<0.001
Sex	1.00	16.16	6.58	0.021
Temporal occurrence	1.00	17.00	7.77	0.013
MDS*Sex	1.00	16.34	0.62	0.444
MDS*Temporal occurrence	1.00	17.00	11.41	0.004
Sex*Temporal occurrence	1.00	17.00	1.45	0.245
MDS*Sex*Temporal occurrence	1.00	17.00	1.67	0.213
Submissive				
MDS	1.00	16.29	6.85	0.018
Sex	1.00	16.35	2.35	0.145
Temporal occurrence	1.00	17.00	8.38	0.010
MDS*Sex	1.00	16.68	0.29	0.595
MDS*Temporal occurrence	1.00	17.00	1.77	0.200
Sex*Temporal occurrence	1.00	17.00	1.76	0.202
MDS*Sex*Temporal occurrence	1.00	17.00	2.30	0.148

* DF Num: Degree of freedom of numerator; DF Den: Degree of freedom of denominator.

**Fig 2:**
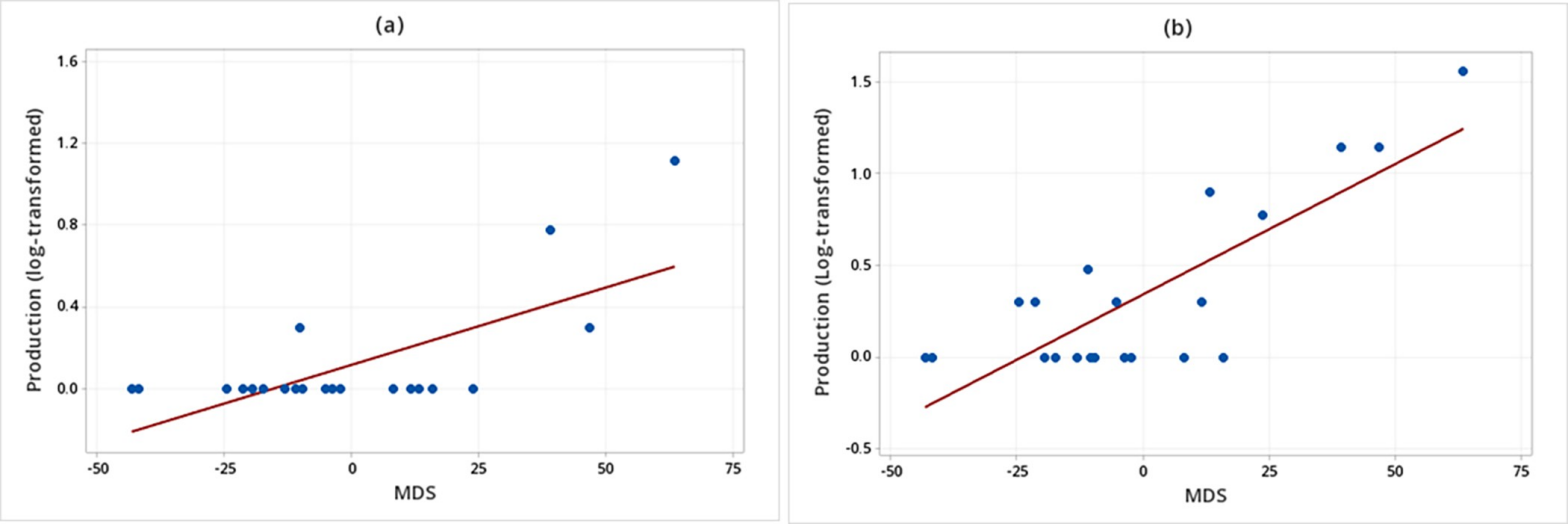
Relationship between the sender’s social rank (MDS) and the production of multimodal signals. (a) White-lipped peccaries (N = 21) were displaying threatening behaviors after a unimodal signal fails. (b) Animals were displaying threatening behaviors without a previous failure.

There was also a significant interaction between the senders’ MDS and the temporal occurrence in the production of multimodal signals when WLPs were displaying threatening behaviors (F_1, 17_ = 11.41, *P* = 0.004) (Table 3). The *post hoc* linear regression analyses showed that the higher the senders’ MDS, the greater the production of multimodal signals when WLPs were displaying threatening behaviors either after or without a previous unimodal signal failure (Production (log-transformed) of multimodal signals in submissive behaviors after a unimodal signal failure = 0.17 + 0.01 MDS (F_1, 19_ = 19.17; R^2^ = 0.60; *P* = 0.022; Fig 2A; Production (log-transformed) of multimodal signals in threatening behaviors without a previous unimodal signal failure = 0.34 + 0.01 MDS (F_1, 19_ = 35.95; R^2^ = 0.64; *P* < 0.001; Fig 2B).

**Fig 3:**
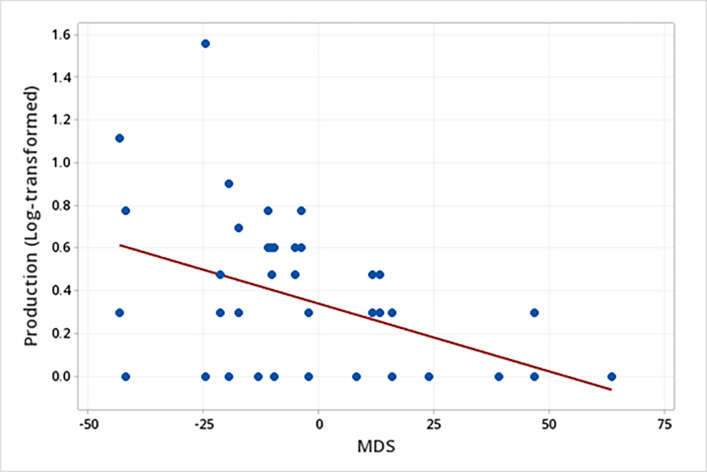
Relationship between the sender’s social rank (MDS) and the production of multimodal signals when displaying submissive behaviors.

Additionally, males used more multimodal signals than females when displaying threatening behaviors (males: mean log-transformed = 0.3, SE = 0.1; females: mean log-transformed = 0.1, SE = 0.1; F_1, 16.16_ = 6.58, *P* = 0.021) (Table 3). Moreover, there was also a relationship between the sender’s MDS and the production of multimodal signals by white-lipped peccaries (N = 21) when displaying submissive behavior, regarding the temporal occurrence (F_1, 16,29_ = 6.85, *P* = 0.018) (Table 3). The *post hoc* linear regression analysis showed that the lower the sender’s MDS, the higher the production of multimodal signals by WLPs when displaying submissive behaviors (Production (log-transformed) of multimodal signals in submissive behaviors = 0.34 – 0.01 MDS (F_1, 41_ = 11.04; R^2^ = 0.22; *P* = 0.002) (Fig 3).

### Individual factors influencing the production of unimodal and multimodal signaling

**Fig 4:**
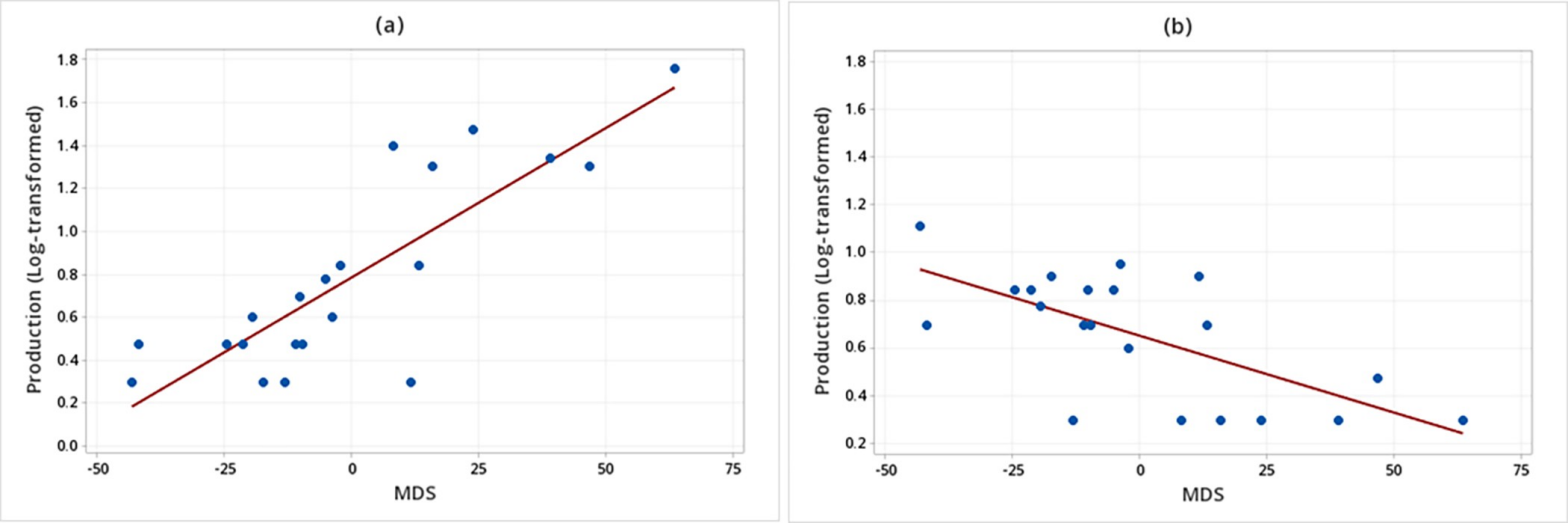
Relationship between the sender’s social rank (MDS) and the production of multimodal signals. (a) White-lipped peccaries (N = 21) were displaying threatening behaviors. (b) Animals were displaying submissive behaviors.

There were significant interactions between the behaviors and the sender’s social rank (MDS) in the production of both unimodal signals (F_1, 34_ = 8.39, *P* = 0.007) and multimodal signals (F_1, 34_ = 34.97, *P* < 0.001) (Table 4). The *post hoc* linear regression analyses, however, showed no significant relationships between the sender’s MDS in the production of unimodal signals when WLPs were displaying either threatening (F_1, 19_ = 2.80, R^2^ = 0.08, *P* = 0.107) or submissive (F_1, 19_ = 1.91, R^2^ = 0.04, *P* = 0.181) behaviors. While regarding the production of multimodal signals, we verified that the higher the sender’s MDS, the greater the production of multimodal signals when WLPs were displaying threatening behaviors; whereas the lower the sender’s MDS, the higher the production of multimodal signals when displaying submissive behaviors (Production (log-transformed) of multimodal signals in threatening behaviors = 0.36 + 0.01 MDS (F_1, 19_ = 35.54; R^2^ = 0.65; *P* < 0.001; Fig 4A; Production (log-transformed) of multimodal signals in submissive behaviors = 0.65 – 0.01 MDS (F_1, 19_ = 15.25; R^2^ = 0.42; *P* = 0.001; Fig 4B).

**Table 4:** Effect of sender’s social rank (modified dominance David’s scores–MDS), sex, and behavior (threatening and submissive) in the production of unimodal and multimodal signals by white-lipped peccaries (N = 21).

Term	DF Num*	DF Den*	F Value	*P*
Unimodal				
MDS	1	34	0.69	0.414
Sex	1	34	0.11	0.747
Behavior	1	34	26.75	<0.001
MDS*Sex	1	34	1.71	0.199
MDS*Behavior	1	34	8.39	0.007
Sex*Behavior	1	34	7.99	0.008
MDS*Sex*Behavior	1	34	0.17	0.681
Multimodal				
MDS	1	34	8.28	0.007
Sex	1	34	1.05	0.312
Behavior	1	34	3.08	0.088
MDS*Sex	1	34	0.56	0.461
MDS*Behavior	1	34	34.97	<0.001
Sex*Behavior	1	34	0.73	0.399
MDS*Sex*Behavior	1	34	0.01	0.951

* DF Num: Degree of freedom of numerator; DF Den: Degree of freedom of denominator.

**Fig 5:**
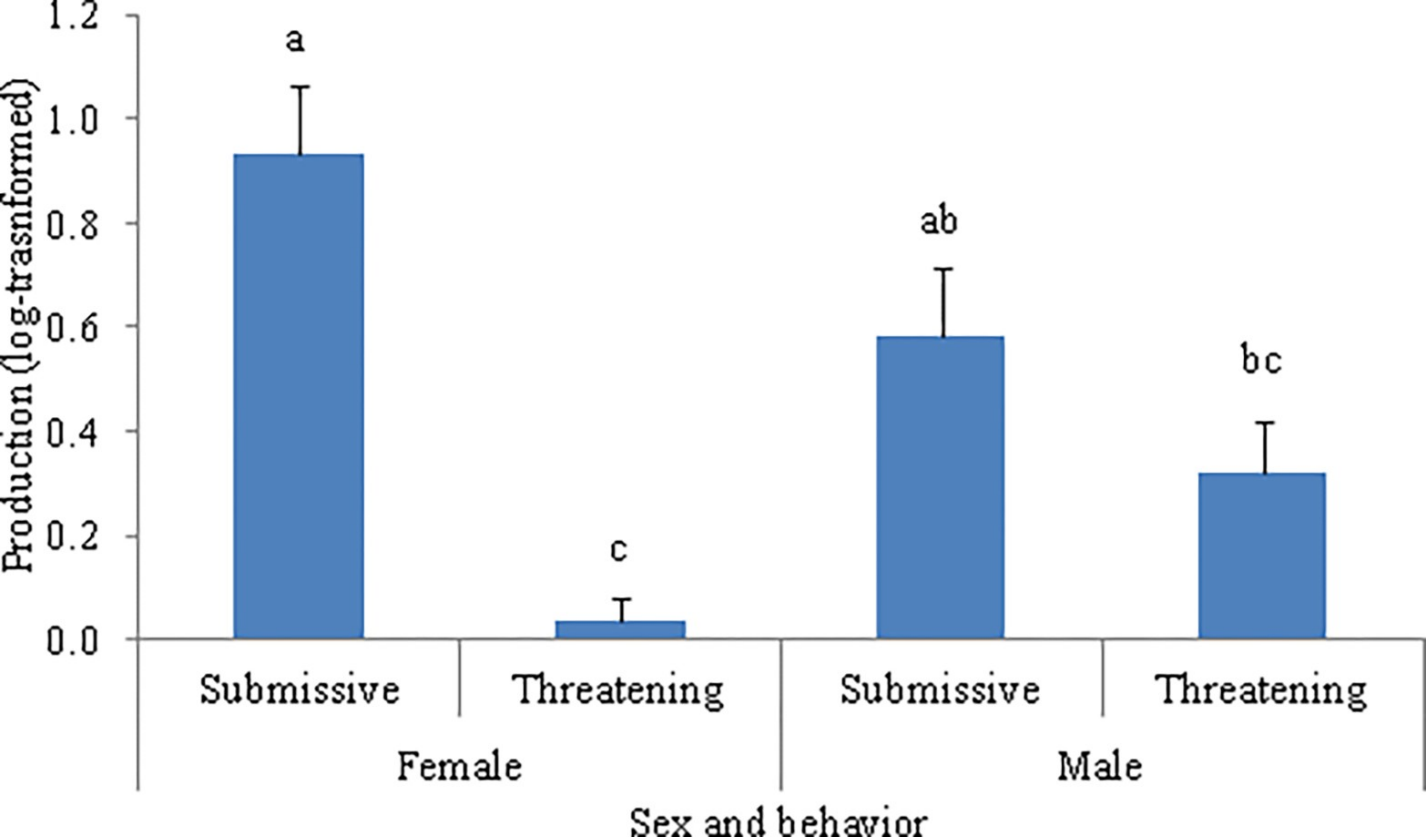
Mean (log-transformed) production of unimodal signals by white-lipped peccaries (N = 21). The female and male white-lipped peccaries could be displaying either threatening or submissive behaviors. Error bars denote SE of means. Different letters above bars represent a significant difference (*P* < 0.05).

As predicted, there was no effect of the sender’s sex on the production of unimodal signals (F_1, 34_ = 0.11, *P* = 0.747) or multimodal signals (F_1, 34_ = 1.05, *P* = 0.312) (Table 4). However, there was a significant interaction between the sender’s sex and the behavior displayed in the production of unimodal signals (F_1, 34_ = 7.99, *P* = 0.008) (Table 4). The *post hoc* Tukey tests showed that females produced more unimodal signals when displaying submissive than threatening behaviors, whereas males did not differ in the production of unimodal signals when displaying threatening or submissive behaviors (Fig 5).

### Individual factors influencing the responses to unimodal and multimodal signaling

**Fig 6:**
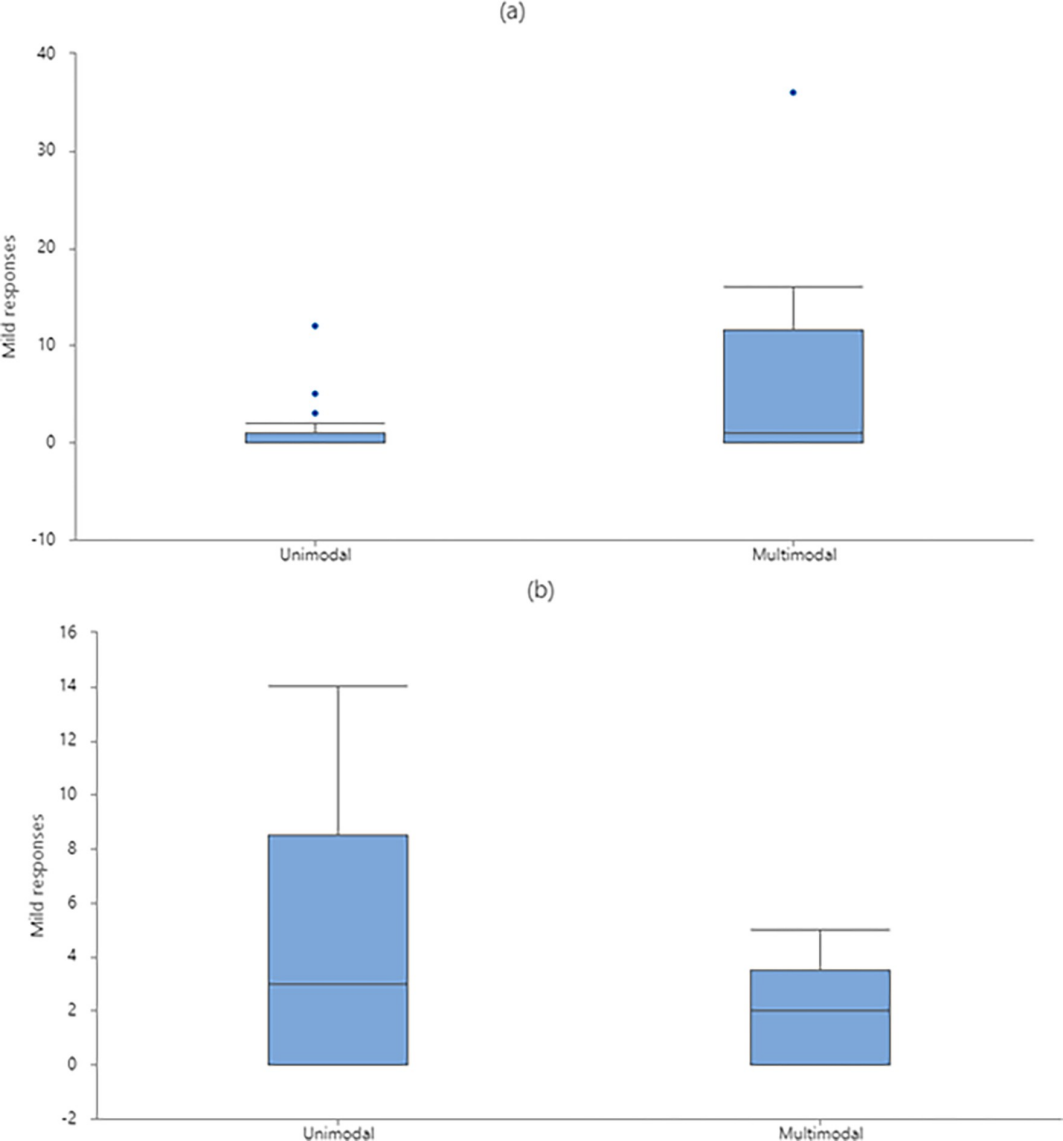
Occurrences of non-aggressive responses to unimodal and multimodal signals produced by white-lipped peccaries (N = 21). (a) Animals were displaying threatening behaviors. (b) Animals were displaying submissive behaviors. Box-and-whisker diagram shows the smallest and largest observations (except outlier(s); box) and lower quartile, median, upper quartile (whiskers). Points represent outliers.

**Fig 7:**
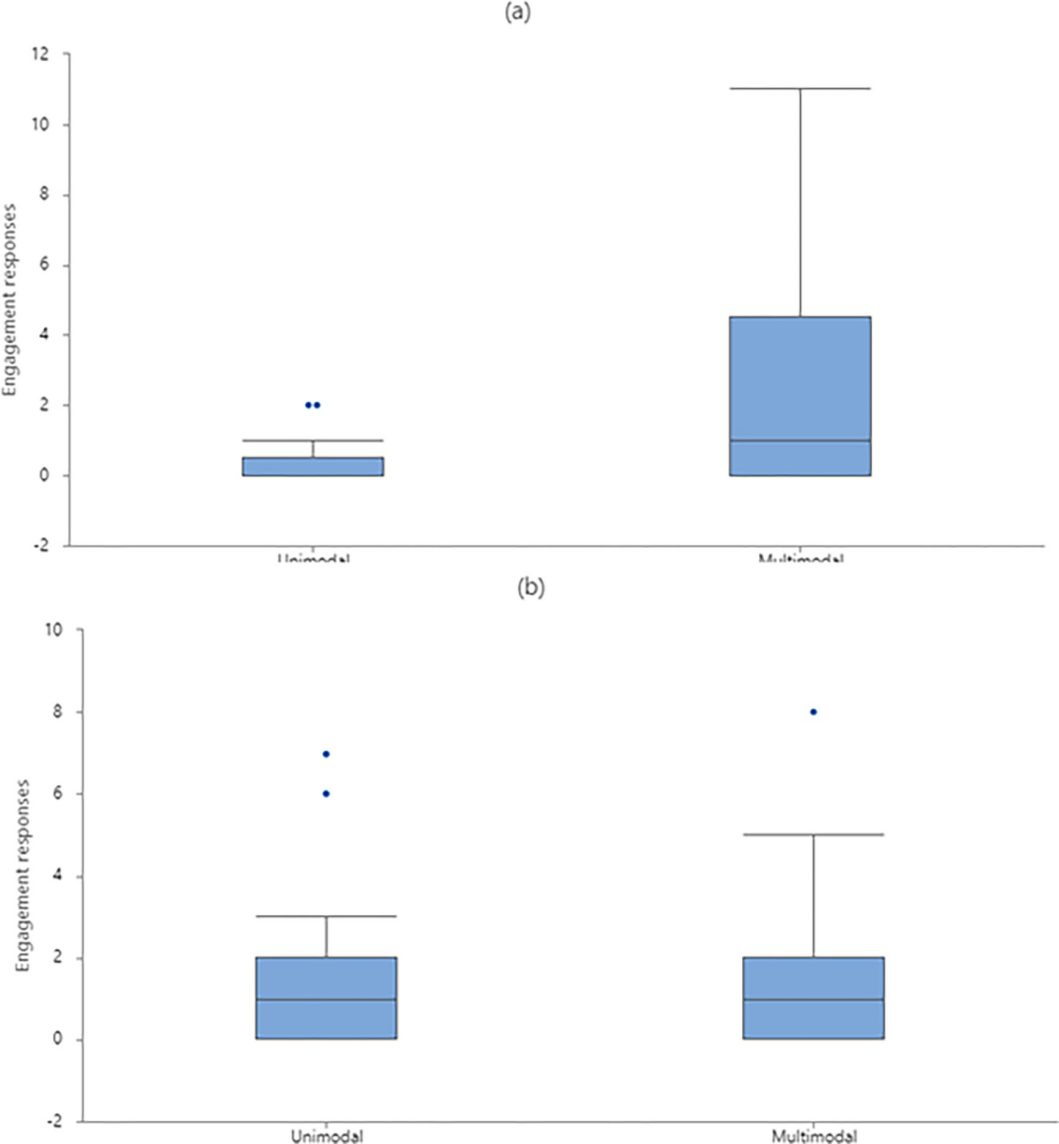
Occurrences of engagement responses to unimodal and multimodal signals produced by white-lipped peccaries (N = 21). (a) Animals were displaying threatening behaviors. (b) Animals were displaying submissive behaviors. Box-and-whisker diagram shows the smallest and largest observations (except outlier(s); box) and lower quartile, median, upper quartile (whiskers). Points represent outliers.

Multimodal signals elicited more non-aggressive responses than did the unimodal signals when WLPs were displaying threatening behaviors (Wilcoxon signed-ranks test, *Z* = 2.35, *P =* 0.019, two-tailed) (Fig 6A); whereas the opposite occurred when displaying submissive behaviors (Wilcoxon signed-ranks test, *Z* = 2.07, *P =* 0.039, two-tailed) (Fig 6B). However, when WLPs were displaying threatening behaviors, multimodal signaling elicited more engagement responses than did the unimodal signals (Wilcoxon signed-ranks test, *Z* = 2.67, *P =* 0.008, two-tailed) (Fig 7A); whereas no such difference was found when displaying submissive behaviors (Wilcoxon signed-ranks test, *Z* = 0.14, *P =* 0.887, two-tailed) (Fig 7B).

**Fig 8:**
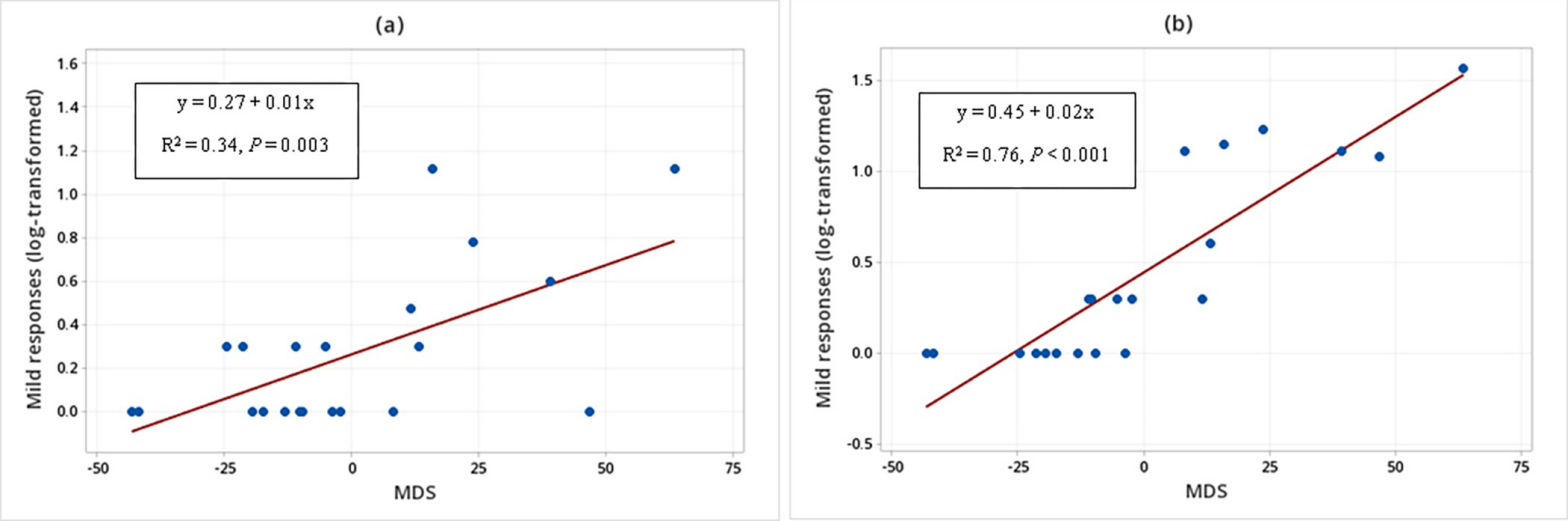
Relationship between the sender’s social rank (MDS) and the occurrence of non-aggressive responses (log-transformed). (a) Occurrence of non-aggressive responses to unimodal signals. (b) Occurrence of non-aggressive responses to multimodal signals, when white-lipped peccaries (N = 21) were displaying threatening behaviors.

There was a significant interaction between the sender’s MDS and signal type in the occurrence of non-aggressive responses when WLPs were displaying threatening behaviors (F_1, 17_ = 11.75, *P* = 0.003) and submissive behaviors (F_1, 17_ = 5.64, *P* = 0.030) (Table 5). The *post hoc* linear regression analyses showed that the higher the sender’s MDS the greater the occurrence of non-aggressive responses whether the WLPs produced unimodal or multimodal signals when displaying threatening behaviors (Non-aggressive responses (log-transformed) to unimodal signals produced when displaying threatening behaviors = 0.37 + 0.01 MDS (F_1, 19_ = 11.50; R^2^ = 0.34; *P* = 0.003; Fig 8A; Non-aggressive responses (log-transformed) to multimodal signals produced when displaying threatening behaviors = 0.45 + 0.02 MDS (F_1, 19_ = 65.71; R^2^ = 0.76; *P* < 0.001; Fig 8B).

**Table 5:** Effect of the sender’s social rank (modified dominance David’s scores–MDS), sex, and signal type (unimodal and multimodal signal) in the occurrences of non-aggressive responses to threatening and submissive behaviors by white-lipped peccaries (N = 21).

Term	DF Num*	DF Den*	F Value	*P*
Threatening				
MDS	1	16.62	33.54	<0.001
Sex	1	16.72	1.93	0.183
Signal type	1	17.00	4.93	0.040
MDS*Sex	1	16.99	0.01	0.935
MDS* Signal type	1	17.00	11.75	0.003
Sex* Signal type	1	17.00	1.16	0.297
MDS*Sex* Signal type	1	17.00	2.25	0.152
Submissive				
MDS	1	17	3.67	0.072
Sex	1	17	0.01	0.907
Signal type	1	17	5.64	0.030
MDS*Sex	1	17	0.01	0.942
MDS* Signal type	1	17	0.35	0.561
Sex* Signal type	1	17	5.28	0.035
MDS*Sex* Signal type	1	17	2.56	0.128

* DF Num: Degree of freedom of numerator; DF Den: Degree of freedom of denominator.

**Fig 9:**
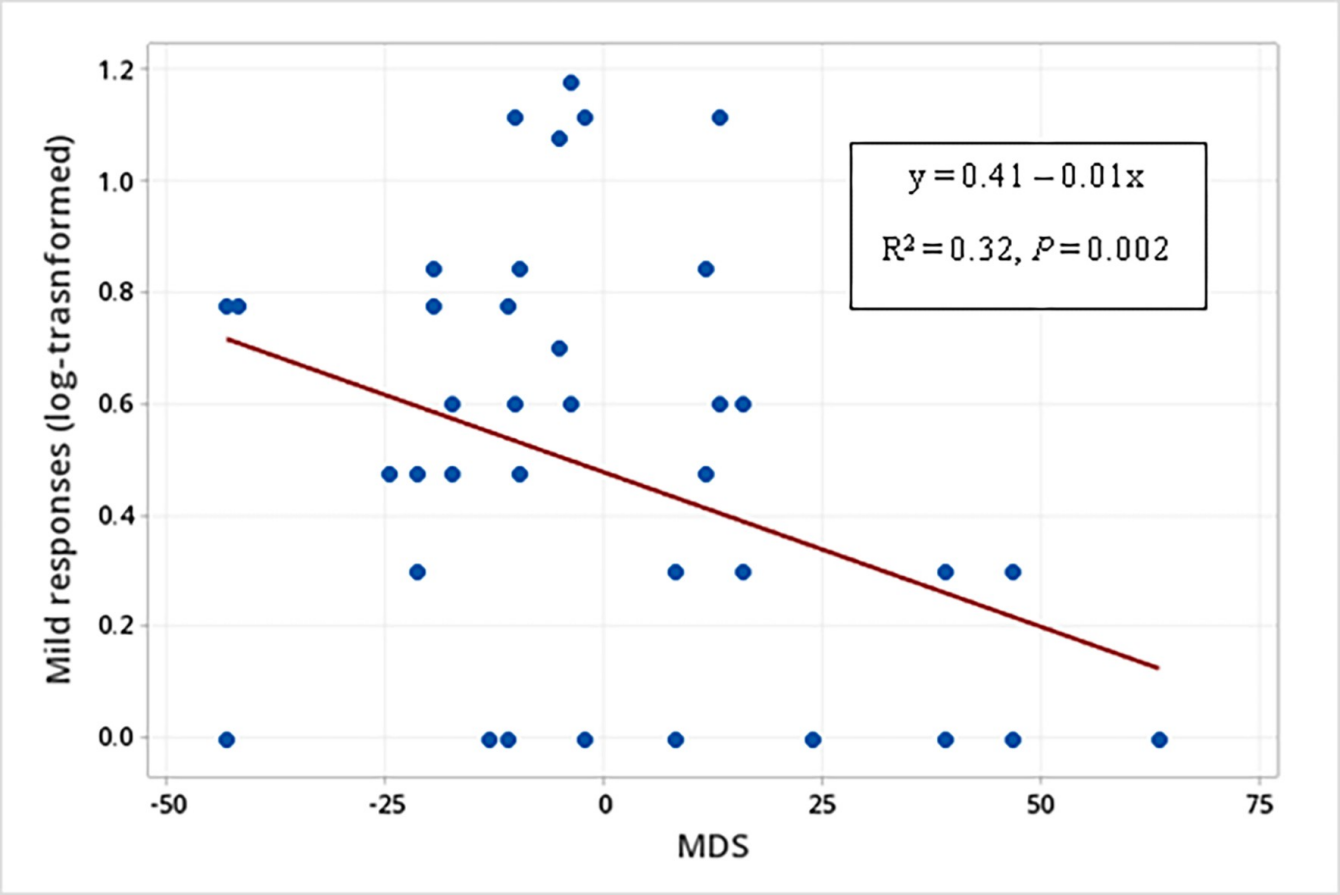
Relationship between the sender’s social rank (MDS) and the occurrence of non-aggressive responses (log-transformed). Occurrence of non-aggressive responses to multimodal signals when white-lipped peccaries (N = 21) were displaying submissive behaviors.

The *post hoc* linear regression analyses, however, showed no relationship between the sender’s MDS and the occurrence of non-aggressive responses to unimodal signals produced when WLPs were displaying submissive behaviors (F_1, 19_ = 1.28, R^2^ = 0.01, *P* = 0.272), whereas the lower the sender’s MDS, the higher the occurrence of non-aggressive responses to multimodal signals produced when WLPs were displaying submissive behavior (Non-aggressive responses (log-transformed) to multimodal signals produced when displaying submissive behavior = 0.41 – 0.01 MDS (F_1, 19_ = 13.00; R^2^ = 0.38; *P* = 0.002) (Fig 9).

**Fig 10:**
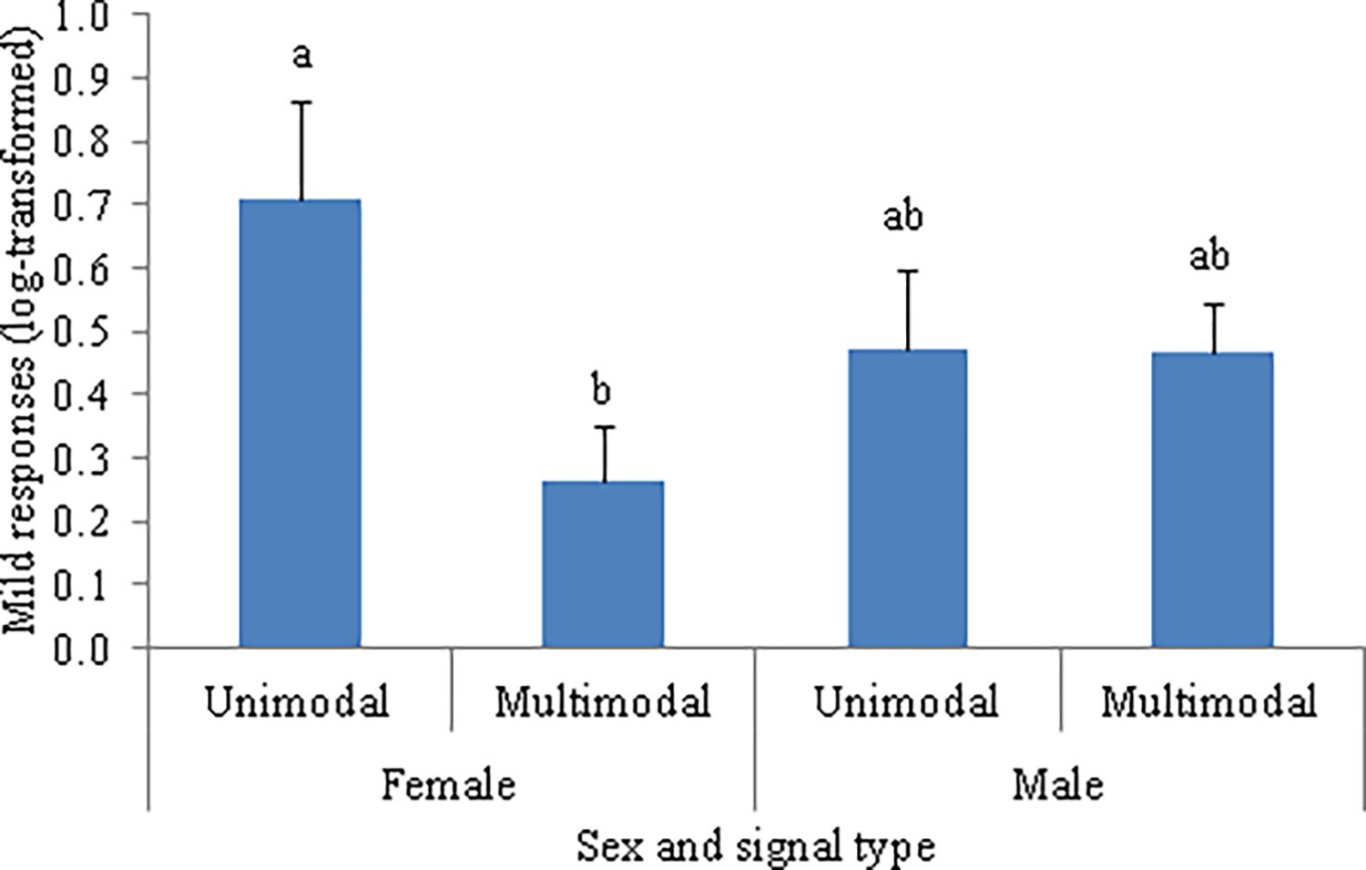
Mean (log-transformed) number of occurrences of non-aggressive responses to unimodal and multimodal signals. The female and male white-lipped peccaries were displaying submissive behaviors. Error bars denote SE of means. Different letters above bars represent a significant difference (*P* < 0.05).

As predicted, there was no effect of the sender’s sex in the responses to threatening behaviors (F_1, 16.72_ = 1.93, *P* = 0.183) or submissive behaviors (F_1, 17_ = 0.01, *P* = 0.907) (Table 5). There was, however, a significant interaction between the sender’s sex and the signal type on the occurrences of non-aggressive responses of WLPs when displaying submissive behaviors (F_1, 17_ = 5.28, *P* = 0.035) (Table 6). The *post hoc* Tukey tests showed that the females showed fewer non-aggressive responses to multimodal signals compared to unimodal signals produced when WLPs were displaying submissive behaviors, whereas the non-aggressive responses to unimodal and multimodal signals did not differ between females and males (Fig 10).

**Table 6:** Effect of sender’s social rank (modified dominance David’s scores–MDS), sex, and signal type (unimodal or multimodal) on the occurrences of engagement responses to threatening and submissive behaviors by white-lipped peccaries (N = 21).

Term	DF Num*	DF Den*	F Value	*P*
Threatening				
MDS	1	33.82	10.63	0.003
Sex	1	33.92	0.32	0.575
Signal type	1	33	12.42	0.001
MDS*Sex	1	33.65	0	0.947
MDS* Signal type	1	33	11.96	0.002
Sex* Signal type	1	33	0.04	0.853
MDS*Sex* Signal type	1	33	0.29	0.591
Submissive				
MDS	1	34	9.75	0.004
Sex	1	34	1.91	0.176
Signal type	1	34	0.27	0.606
MDS*Sex	1	34	1.64	0.208
MDS* Signal type	1	34	0.07	0.791
Sex* Signal type	1	34	5.68	0.023
MDS*Sex* Signal type	1	34	0.97	0.333

* DF Num: Degree of freedom of numerator; DF Den: Degree of freedom of denominator.

**Fig 11:**
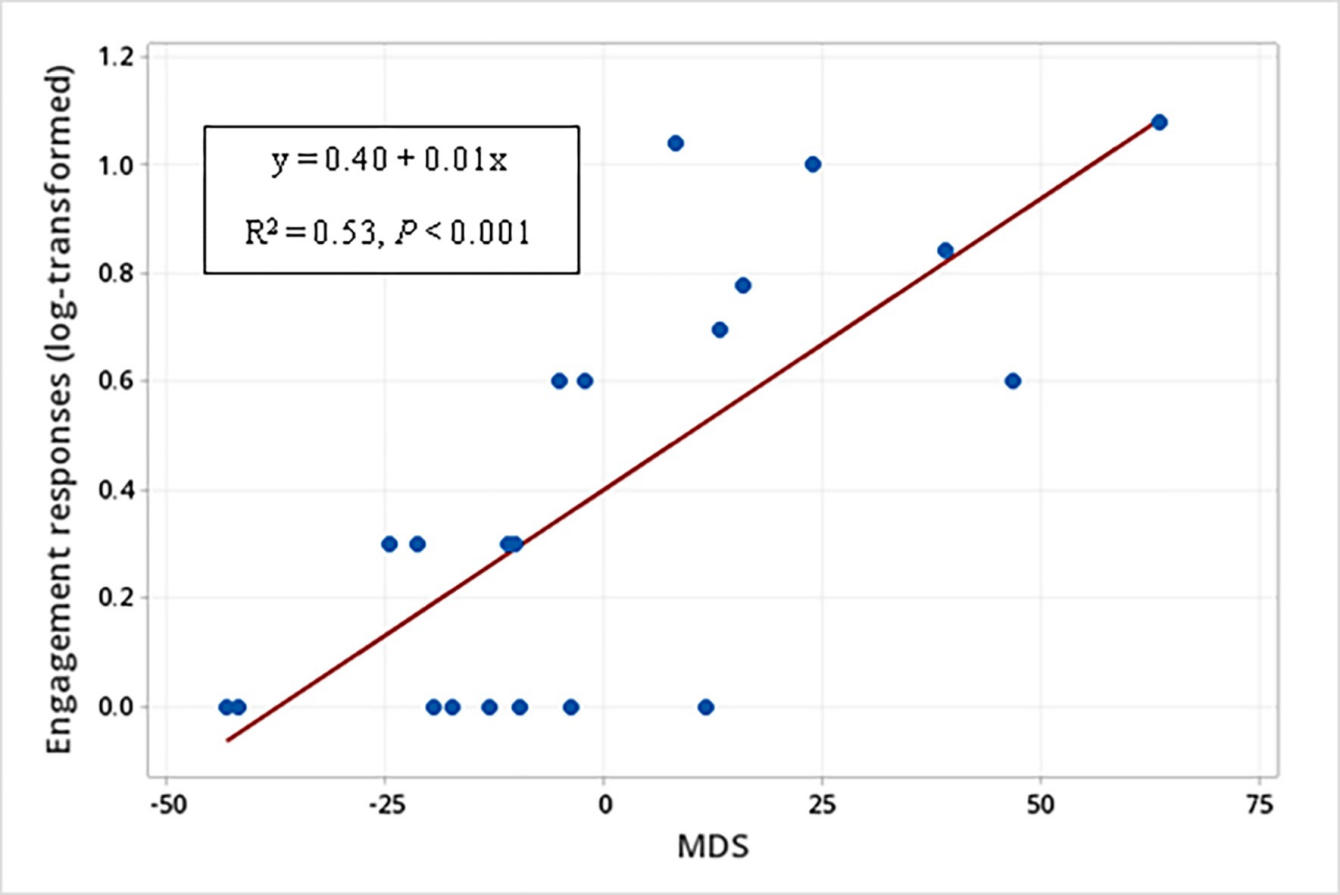
Relationship between the sender’s social rank (MDS) and the occurrence of engagement responses (log-transformed). Occurrence of engagement responses to multimodal signals when white-lipped peccaries (N = 21) were displaying threatening behaviors.

There was also a significant interaction between the sender’s MDS and the signal type produced in the occurrence of engagement responses when WLPs were displaying threatening behaviors (F_1, 33_ = 12.42, *P* = 0.001) (Table 6). The *post hoc* linear regression analyses showed no relationship between the sender’s MDS and the occurrence of engagement responses when WLPs produced unimodal signals when displaying threatening behaviors (F_1, 19_ = 1.28, R^2^ = 0.01, *P* = 0.272); whereas regarding the engagement responses we verified that the higher the sender’s MDS, the greater the occurrence of engagement responses when WLPs produced multimodal signals when displaying threatening behaviors (Engagement responses (log-transformed) to multimodal signals produced when displaying threatening behaviors = 0.40 + 0.01 MDS (F_1, 19_ = 23.27; R^2^ = 0.53; *P* < 0.001) (Fig 11).

**Fig 12:**
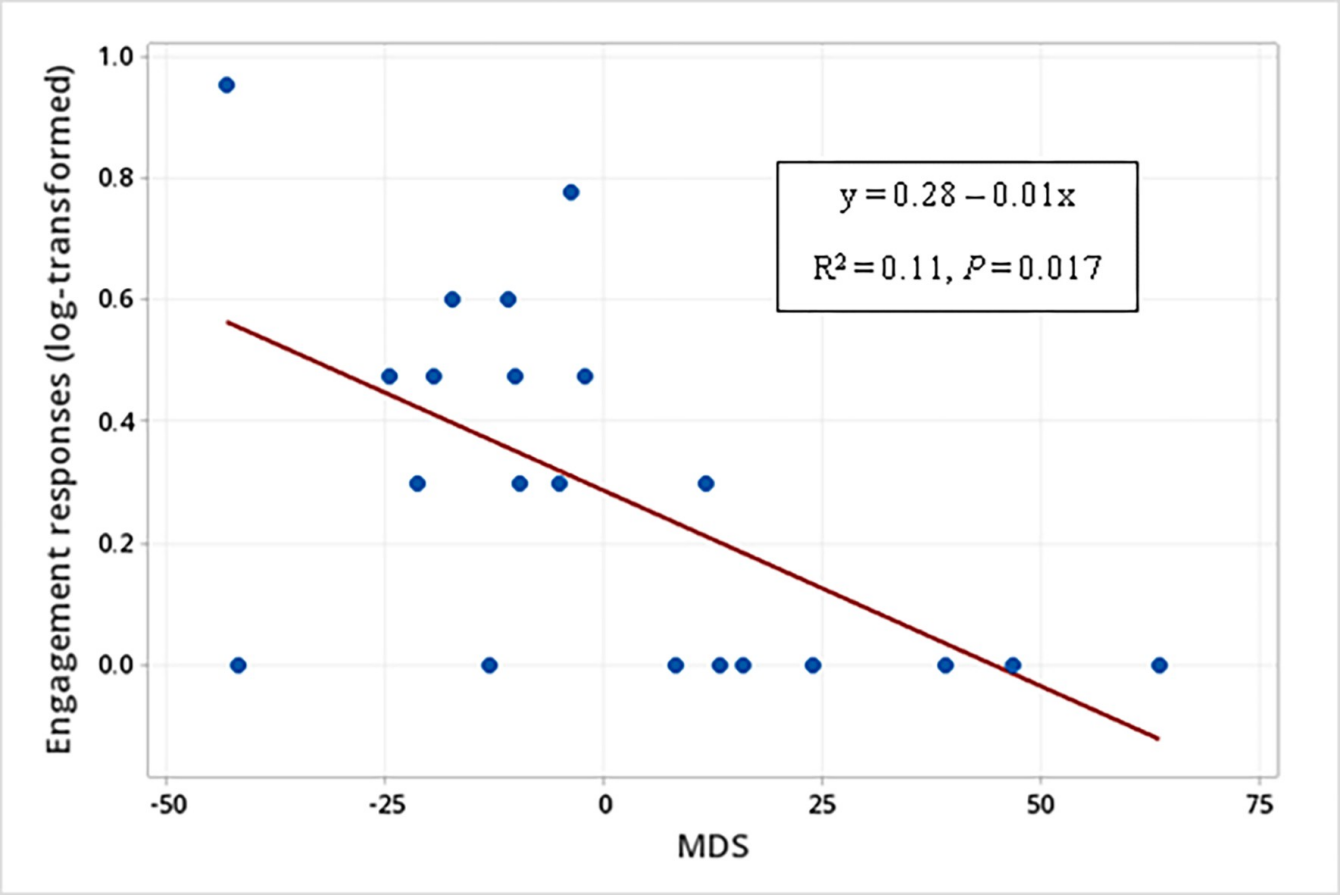
Relationship between the sender’s social rank (MDS) and the occurrence of engagement responses (log-transformed). Occurrence of engagement responses to unimodal and multimodal signals produced when white-lipped peccaries (N = 21) were displaying submission behaviors.

The sender’s MDS also affected the occurrence of engagement responses when WLPs were displaying submissive behavior (F_1, 34_ = 9.75, *P* = 0.004) (Table 6). The higher the sender’s MDS, the lower the occurrence of engagement responses when WLPs produced either unimodal signals or multimodal signals when displaying submissive behavior ((Non-aggressive responses (log-transformed) to multimodal signals produced when displaying submissive behaviors = 0.28 – 0.01 MDS (F_1, 40_ = 6.05; R^2^ = 0.11; *P* = 0.017) (Fig 12).

**Fig 13:**
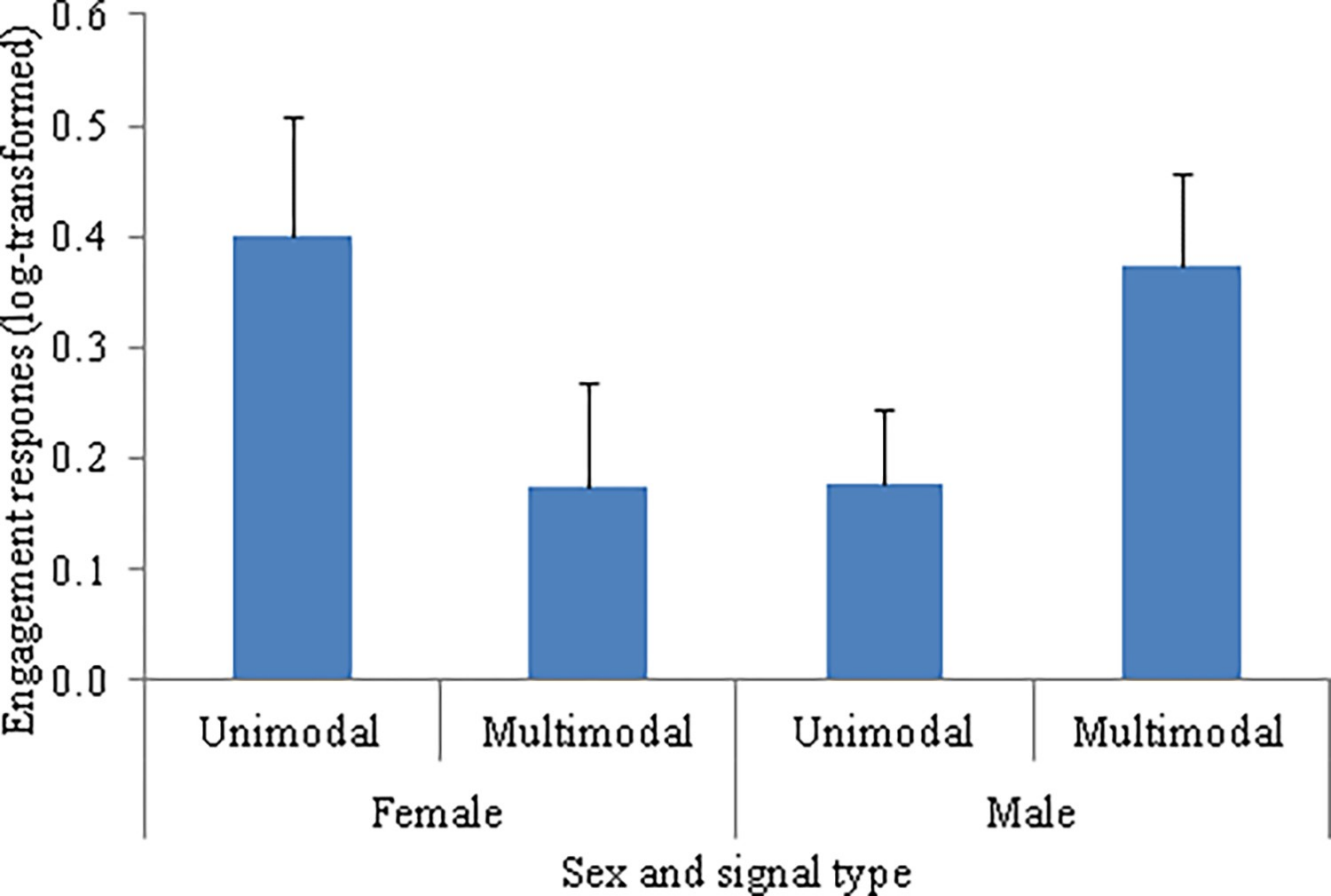
Mean (log-transformed) number of engagement responses to unimodal and multimodal signals. The female and male white-lipped peccaries were displaying submissive behaviors. Error bars denote SE of means.

Furthermore, there was a significant interaction between the sender’s sex and the signal type produced in the occurrences of engagement responses of WLPs when displaying threatening behaviors (F_1, 17_ = 5.668, *P* = 0.023) (Table 6). However, the *post hoc* Tukey tests showed that the engagement responses to unimodal and multimodal signals produced when WLPs were displaying submissive behaviors did not differ between females and males (Fig 13).

## Discussion

The production of multimodal signals by WLPs occurs more often than that of unimodal signals, and independently of the failure of a previous unimodal signal. This result supports the ‘multiple messages’ or ‘complementarity’ hypothesis [9–14] to explain the function of multimodal signaling during agonistic interactions in WLPs. Therefore, by adding novel and non-redundant information in agonistic interactions, such as the size of the sender and its presumed intent, the message will be better understood [12,14]. [12,14] As we expected, our results have shown that individuals with more submissive social status produce more multimodal signals when displaying submission. As the use of multimodal signals leads the sender to expend more time and energy [4,5,12], it would be reasonable to assume that that even so it is advantageous for subordinate WLPs to produce more multimodal signals when signaling submission. However, in contrast to our prediction, the production of multimodal signals when displaying submission usually elicited more engagement (dominance-submission) responses than did the unimodal signals. We also verified that the lower the sender’s social rank, the greater the occurrence of engagement responses to multimodal signals when displaying submission. Thus, intriguingly, the lower-ranking WLPs do not seem to benefit from multimodal signals that would apparently be produced to reinforce their submission. On the contrary, quite interestingly and unexpectedly, the production of multimodal signals to demonstrate submission seems to serve as a trigger for a more aggressive response from the opponent. This response could be explained by the signaling dominance of conspecifics eavesdroppers (see below).

Our results also show that the higher the sender’s social rank, the greater the production of multimodal signals when displaying a threat. Moreover, the production of multimodal signals usually elicited a higher number of non-aggressive responses than did the unimodal signals when WLPs were displaying threatening behaviors. The effectiveness of social-status signaling is advantageous to the top-ranking WLPs, because besides allowing conflict resolution without costly fights, this avoids unnecessary expenditure of energy during the competition for limited resources as well. Among other consequences, this saving in energy can avoid delaying the movements of WLP groups that travel up to 10 km daily under natural conditions [49]. Furthermore, the production of multimodal signals by top-ranking WLPs when displaying a threat seems to be associated with signaling dominance not only to their direct opponent, but also to conspecifics eavesdroppers [2], as seen in non-human primates. Among free-ranging chimpanzees, visual and tactile signals are perceived only by the immediate audience. However, by incorporating auditory signals, the same individual can be perceived by others further away [24]. WLPs show third-party interventions in aggressive disputes, in which more dominant individuals lean on each other to end a conflict with a subordinate [53]. Thus, it is reasonable to assume that, by incorporating auditory signals into the visual and/or tactile signals when signaling a threat, top-ranking WLPs may be trying to attract the attention of allies to help and to put an end to the quarrel. Further studies, however, must be done to test this hypothesis more accurately.

As far as we know, few studies have explored the use of multimodal communication as a way to assert the sender’s dominance status. Apparently, dominant eland bulls (*Tragelaphus orix*) use multimodal signals to emphasize their social status in reproductive periods [26]. In this species, which lives in mixed-sex herds like WLPs, the bull’s facial ornamentation (dark facemask and large face-brushes) together with a knee-click sound, which has distinct acoustic characteristics that encode the sender’s skeletal size and muscular development, reveal the individual’s social status [26]. Furthermore, it was found among male chimpanzees that the higher the social rank of the individuals, the greater the use of combined visual and auditory signals [24]. Although both studies, which were done observing free-ranging animals, have pointed out the production of multimodal signals to express the sender’s social status, there are no systematic analyses like those presented here. Only one study has examined the relationship between the sender’s social rank and the production of, and responses to, multimodal signals in wild chimpanzees. However, in contrast to what we described here for WLPs, no such relationships were found in chimpanzees [64].

There was a higher production of multimodal signals compared to unimodal signals in agonistic interactions between captive WLPs, in contrast to what was found in wild chimpanzees [64]. One can argue that captive conditions, which favor a greater proximity between group members, could explain these results. However, multimodal signal production is relatively low in captive bonobos [57]. The greater production of multimodal signals compared to unimodal signals by WLPs could be explained by the relatively small groups observed in the present study in comparison to the larger groups found in the Amazon region [48,49]. However, as commented before, WLPs can also be found in much smaller groups of similar size to those studied here, due to seasonal food shortages and overhunting [50–52]. Additionally, as highlighted by Leonardo et al. [53], it is almost impossible to study the social behavior of free-ranging WLPs due to high hunting pressure, which developed an effective anti-predator avoidance strategy against humans [66]. Such characteristics do not allow for habituation to human presence, which is essential to conduct this type of study. Therefore, it would be very interesting to conduct further studies on larger groups of captive WLPs, housed in larger areas and with thicker vegetation, similar to that found in their natural environment [48], to confirm all the results described here.

Olfactory signals may also play a fundamental role in social status signaling of WLPs. The substances excreted by the odoriferous dorsal gland [44] may be used for recognition of family members and respective kinship, as already suggested [53]. Thus, it is possible that WLPs combine olfactory signals with visual, auditory, and/or tactile signals. However, in the present study there were no technical conditions in which to analyze the role of olfactory signals, and they still need to be tested for better comprehension of the maintenance of WLP societies. Moreover, as the social ranking is dynamic over time [28], further studies that join those changes in dynamics in the individuals’ social rank would indicate whether unimodal or multimodal signal production is affected by these ranking changes. Despite all these considerations, we hope that our results stimulate other researchers to increase knowledge on how non-human social vertebrates use multimodal communication to express their social status, avoiding conflicts and thereby promoting group cohesion.

## Conclusion

Our findings support the ‘multiple messages’ hypothesis to explain the function of multimodal signaling during agonistic interactions in white-lipped peccaries. Thus, they produce multiple signals that allow the transfer of independent information, such as the size of the sender and its intention, leading to an increase in the information content that is sent to the receiver. Additionally, both the production of, and responses to, multimodal signals are related to the sender’s social rank. We verified that when displaying a threat, the production of multimodal signals often elicited a larger number of non-aggressive responses than did the unimodal signal behaviors; this, in turn, allows conflict resolution without costly fights, avoiding unnecessary expenditure of energy during the competition for limited resources. These results allow us to suggest that the production of multimodal signals may have a key role in mitigating conflict and thus promoting group cohesion among white-lipped peccaries.
